# Antimalarial treatment by health care providers in Port Harcourt, Nigeria

**DOI:** 10.1186/1475-2875-11-S1-P28

**Published:** 2012-10-15

**Authors:** Omotavo O Ebong, Erne O Asuguo, Chijioke A Nwauche, lyeopu M Siminialayi, Ijeoma H Ogbuehi, Mercy F Ajienka

**Affiliations:** 1 Center for Malaria Research and Phytomedicine; University of Port Harcourt, Rivers State, Nigeria

## Background

In Nigeria, malaria accounts for 60% of outpatient visits, 30% hospitalization, and is estimated to be responsible for about 11% of overall maternal mortality, 25% of infant mortality, and 30% of under-five mortality [1]. The disease is particularly virulent among pregnant women and the under-five years of age, due to their low levels of immunity. It impedes economic growth and keeps households in poverty. Lack of access to diagnostic testing before treatment is one of the weaknesses in the management of malaria in Nigeria [2]. This study examines the treatment practice for malaria among health care providers (HCPs) in Port Harcourt.

## Materials and methods

This was a cross-sectional study among HCPs, and data collection was by use of pre-validated questionnaires and in-depth interviews. The data was analyzed using SPSS Version 17.

## Results

A total of 273 HCPs (doctors, nurses, pharmacists, community health workers (CHWs) and private medicine vendors (PMVs)) were randomly selected from health care facilities in Port Harcourt. Of the HCPs, 100% of doctors & pharmacists; 89.6% nurses; 33.3% PMVs; and 25% CHWs are aware of the World Health Organizations(WHO) treatment guidelines. The ACTs (69.2%) and sulphadoxine / pyrimethamine (7.7%) were the most prescribed drugs for uncomplicated malaria in children. Other drugs prescribed were: Chloroquine, Quinine, and Artesunate (group 1), 5.1%; and Pyrimethamine and Paracetamol (group 2), 2.6%. For severe malaria in children, Quinine (46.2%), the ACTs (20.5%) and intravenous artemether (12.8%) were mostly used. The other drugs prescribed were those in groups 1 and 2 above. For uncomplicated malaria in adults, the ACTs (66.7%) and sulphadoxine /pyrimethamine (17.9%) were most prescribed in addition to the other drugs in groups 1 and 2. For severe malaria in adults, Quinine (46.2%), the ACTs (20.5%) and intravenous artemether (12.8%) were mostly prescribed. For pregnant women, sulphadoxine /pyrimethamine (76.9%), the ACTs (10.3%), Artesunate (7.7%) and Quinine (5.1%) were mostly prescribed. Regarding adherence to WHO treatment guidelines, only 44.3% of HCPs [doctors, 52.1%; Nurses, 23.1%; Pharmacists, 11.6%; PMVs, 7.4% and CHWs, 6.0%] used diagnostic testing before treatment. Proximity to a good laboratory, laboratory costs, and availability of diagnostic tools are major factors that influence HCPs’ decisions in carrying out proper diagnosis before treatment.

## Conclusion

This study shows that, while the ACTs are widely used for the treatment of malaria in Nigeria, a larger proportion of the treatment is not based on diagnostic evaluation. Many HCPs recognize that diagnostic testing should precede treatment, but do not have the required facilities for it.
